# ‘Like flesh and a nail’: rethinking the nexus of familial ties and armed conflict

**DOI:** 10.1177/13540661251323177

**Published:** 2025-03-18

**Authors:** Hanna Ketola, Maria O’Reilly

**Affiliations:** Newcastle University, UK; Leeds Beckett University, UK

**Keywords:** Family, war, female combatants, armed groups, feminism, social ties

## Abstract

This article advances a feminist theorization of the critical nexus between family and armed conflict. It does so by examining the relationship between familial ties and women’s participation in fighting forces. We focus on two key questions: What are the familial ties that are constituted through conditions of war? And how do these ties shape women’s participation in armed groups, in various forms? Critical IR and feminist scholarship recognize that family sustains war symbolically and materially. Yet, what is missing is a theoretical conceptualization of the relationship between the diverse ties that constitute family in contexts of war and women’s participation in armed groups. Our novel framework – of *militarized familial ties* – conceptualizes familial ties as affective bonds that both emerge through and are transformed by war’s violence. This dynamic framing allows us, first, to systematically illustrate how familial ties shape key processes pursued by armed groups, including the recruitment and retention of fighters. And second, our framing offers crucial new insights into how the political subjectivities of women fighters intersect with familial ties. We offer a new *typology of militarized familial ties* to illustrate how pre-existing and emergent familial ties both condition, and are conditioned by, women’s participation in armed groups. We demonstrate the wider implications of our theoretical intervention by reflecting on long-term field research conducted in Bosnia and Herzegovina (BiH) and Nepal.

## Introduction

Reflecting on her experience of serving with fellow soldier Lejla in the Army of the Republic of Bosnia & Herzegovina (ARBiH) during the 1992–1995 war, Biljana remarked: ‘This friend of mine . . . used to be like my sister, because I spent more time with her than with my own sisters’. Dil Sari, a political leader in the Maoist movement, revealed the challenges of leaving her young daughter with family members, to participate in a major attack during the 1996–2006 ‘People’s War’ in Nepal: ‘It is a big thing for a mother to decide to leave her child’. She described how her cousin cared for the baby as if she were her sister. These armed conflicts and fighting forces were strikingly different. The ARBiH, created to defend the newly independent Bosnia & Herzegovina against nationalist rebellion, included relatively few women in combat roles. In contrast, the Maoist movement, pursuing the campaign of creating a ‘New Nepal’ encouraged the mass participation of women in its armed wing (the People’s Liberation Army, PLA). Nevertheless, both Biljana and Dil Sari spotlighted the importance of familial ties in shaping their participation in armed struggle, and both women underscored the affective nature of the familial ties that emerged and endured in and through war.

This article advances a feminist theorization of the critical nexus between family and armed conflict. It does so by examining the co-constitutive relationship between familial ties and women’s participation in fighting forces. We focus on two key questions: What are the familial ties that emerge and transform within and through conditions of war? How do these ties shape women’s participation in armed groups, in various forms? In our framing, familial ties encompass attachments that are expressed in familial terms and are thus not reducible to blood relations or narrow conceptualizations of nuclear family (Matarazzo and Baines, 2021; Zharkevich, 2019).^1^ Furthermore, our focus is not on all kinds of familial ties, but specifically on ‘militarized familial ties’ – ties that are emergent from and configured through war’s violence (Ketola, 2023).

In examining the nexus of familial ties and armed conflict, critical IR scholarship has powerfully shown that states draw on family as a gendered and racialized social institution to enable war (Friedman and Ketola, 2024; Hartnett, 2023; McClintock, 1993; Turner, 2020). Feminist IR studies have demonstrated precisely *how* family becomes militarized – entangled with military and political aims (Basham and Catignani, 2018; Enloe, 2000), with recent scholarship extending this analysis to contexts of armed groups and civil wars (Hedström, 2020; Madhani and Baines, 2020). Furthermore, Conflict Studies research recognizes that everyday connections between family members, friends and neighbours are central to understanding the dynamics of armed conflict, including army organization, mobilization and retention (Parkinson, 2023; Shesterinina, 2021; Staniland, 2014).

What is missing in the current scholarship is a deeper understanding of the relationship between, first, the diverse range of ties that constitute family in contexts of war, and second, women’s participation in fighting forces. Some recent Conflict Studies scholarship continues to identify the (heterosexist) family as a key obstacle to the recruitment and deployment of *female* combatants, rather than an enabling mechanism. The potential for family to facilitate, rather than constrain, women’s engagement in armed groups is largely overshadowed when family is *primarily* viewed as a mechanism of patriarchal control (Manekin and Wood, 2020: 1644; cf. Thomas and Wood, 2018: 218). From a different angle, feminist IR scholarship warns that stereotypical and heterosexist associations between women, motherhood and the family realm are still routinely used in mainstream media to downplay the agency and political commitment of female fighters (Åhäll, 2012). There is therefore a valid feminist concern that a gendered association of ‘women’ with ‘family’ in scholarly analysis would similarly thwart the recognition of women’s political subjectivities *as* fighters (Gentry and Sjoberg, 2015; Žarkov, 2007). Yet, this is precisely why we need a feminist critical framework that systematically examines the relationship between familial ties and women’s participation in fighting forces.

To address this lacuna, this article offers a new theoretical framework – of *militarized familial ties* – to capture precisely *how* familial ties *shape*, and are *shaped by*, women’s participation in fighting forces. This involves examining the cultural, material, and affective bases of women’s participation. Our framework builds on feminist theories of the relationality of violence (Baines, 2016; Das, 2006) and diverse feminist scholarship on the cultural and material bases of women’s participation in armed groups (Hedström, 2020; Viterna, 2013; Žarkov, 2007; Friedman, 2018). At its core, our framework views familial ties as distinctly *affective bonds* that emerge and transform within and through armed conflict (Ketola, 2023). This allows us to demonstrate how familial ties can operate as a generative (rather than solely constraining) force, which prompts and shapes women’s participation in fighting forces in distinct ways.

The notion of ‘affective bonds’ captures an emotional investment in the lives of others and relationships to others, including the affective labour that goes into sustaining such attachments (Chisholm and Ketola, 2020; Matarazzo and Baines, 2021). We argue that familial ties *matter* for understanding armed conflict not only because family is a powerful social institution. They also matter because the women participating in armed groups continue to cultivate and affectively invest in these ties and the norms that structure them.

To systematically capture and analyse the co-constitutive relationship between familial ties and women’s participation in armed groups, we construct two frames: ‘familial ties as emergent from war’ and ‘familial ties as transformed by war’. The first captures the types of ties that women’s participation *generates* within and through the armed group, highlighting how these ties may be expressed in familial terms (Zharkevich, 2019). Zooming in on *processes of transformation*, the second frame details how both *emergent* and *pre-existing* familial ties are embedded in, and reconfigured in relation to, wider transformations of gender norms that war affects (Wood, 2008).

Our argument makes distinct contributions to Feminist IR and the broader Conflict Studies scholarship on the nexus of family and armed conflict. First, we offer crucial new insights into feminist theorization of the political subjectivities of women fighters, by capturing the distinct ways in which these subjectivities are crafted through, and pursued in relation to, familial ties. Furthermore, while feminist studies have extensively documented the cultural politics of motherhood (Åhäll, 2012; Gentry and Sjoberg, 2015), our framework broadens the range of familial ties in focus. For example, we show the importance of ‘sisterly ties’ that emerge through participation in armed groups, moving beyond the current emphasis on male bonding and military masculinities in feminist IR literature on armed conflict (MacKenzie, 2015).

Second, we contribute to the wider IR and Conflict Studies literature on social ties that explores how everyday ties condition armed group processes in highly significant ways (Parkinson, 2013; Shesterinina, 2021; Viterna, 2013). While this scholarship is crucially important, it provides only partial theorization of why and how familial ties matter for understanding armed group processes. First, the distinctive quality of familial ties goes missing as these ties are analysed as a component of the realm of ‘everyday ties’ and not conceptualized in relation to family as a gendered institution that is militarized in specific ways (Ketola, 2023). Second, even in the nuanced analyses that integrate gender, the affective dimension of familial ties remains under-theorized. This is partly because the literature focuses on the (important) question of how armed groups *as organizations* strategically shape the realm of the family and the ties that constitute it (Parkinson, 2023). In contrast, our rethinking of familial ties as distinctly affective bonds allows us to capture how familial ties are cultivated and inhabited by women who join armed groups. Building on this, our *typology* of militarized familial ties systematically illustrates how familial ties create contradictory demands for women in fighting forces, with women making decisions regarding whether to join, sustain their participation or leave armed groups vis-à-vis the multiple affective attachments they inhabit and negotiate.

While this article is primarily intended as a theory development piece, we illustrate our theoretical framings through empirical reflections from Bosnia & Herzegovina (BiH) and Nepal, drawing on long-standing field research. We delve deep into narratives of women who joined fighting forces in these two distinct contexts, to systematically identify how women’s participation in armed groups is enabled, conditioned *and* constrained by a dynamic web of familial ties in which women fighters are embedded.

Our paper proceeds as follows: the first two sections focus on building our theoretical framework of *militarized familial ties*, capturing the cultural, material, and affective bases of women’s involvement in fighting forces. First, the section ‘Understanding the Cultural and Material Bases of Women’s Participation in Fighting Forces’, develops our conceptualization of *family* as militarized in contexts of war. Building on this, the section ‘Familial Ties as Affective Attachments’ details our conceptual framing of *familial ties* as affective bonds that emerge and transform in conditions of war. The third section introduces our case studies and methodological approach. The final section is dedicated to our analysis and is divided into three main parts. We begin by focusing on the familial ties that war’s violence generates, examining how new ties emerge within and through women’s participation in fighting forces. Next, we explore how familial ties as affective bonds are reshaped and transformed within the context of armed conflict, which prompts wider shifts in societal gender norms. Finally, we offer a *typology* of militarized familial ties, to systematically capture how familial ties are produced and (re)configured vis-à-vis armed group recruitment and retention processes. We conclude by detailing how our rethinking of familial ties offers new insights into the nexus between family and armed conflict.

## Understanding the cultural and material bases of women’s participation in fighting forces

### Cultural bases of participation

Women’s mobilization into fighting forces is conditioned by gendered, and culturally specific, power relations which shape expectations about women’s roles and behaviours during wartime (Parashar, 2014). Here, we explore the ‘cultural bases of women’s participation in fighting forces’ – to conceptualize how the (heterosexual) family surfaces in the gendered language, symbols, and images surrounding armed conflict, and shapes the capacities of women to participate in armed struggle.

An extensive body of scholarship, on gender and nationalism, highlights how familial ideals play a crucial role in constructing the nation and shaping the social and cultural roles of its members (Žarkov, 2007). Ethno-national discourses, for example, are often deeply conservative in depicting men as heroic warriors and active protagonists of national struggles while women are portrayed in subordinate roles as biological and cultural reproducers of the nation and its traditions (Anthias and Yuval-Davies, 1989). Ethno-nationalist political leaders frequently depict the nation as a patriarchal, heterosexual family, allowing them to assign gendered roles in wartime (McClintock, 1993). In the former Yugoslavia, for example, ethno-nationalist movements portrayed women in passive roles as patriotic mothers, faithful wives and victims of male aggression, to bolster the enlistment of men and public support for war (Mostov, 2012). Yet, women actively engaged in political struggle – both in support of, and resistance to, ethno-nationalism – including by taking up arms (Žarkov, 2007). In besieged Sarajevo, for example, a small yet not insignificant number of women joined the ARBiH in response to the separatist violence unleashed in 1992–1995. Bosnian women fought to defend themselves, their families and neighbours, and to defend BiH as a united, multi-ethnic state (Hadžiahmić, 2011).

Nationalist discourses are, however, articulated differently across contexts. Some movements, for example, may be underpinned by a commitment to social transformation and gender equality. In Nepal, the Communist Party of Nepal-Maoist (CPN-M) explicitly integrated ‘gender equality’ as part of their stated vision for ‘New Nepal’. This involved restructuring the institution of family – for example, through instituting revolutionary marriage within the PLA (Zharkevich, 2019). In this context, the mass recruitment of women into the Maoist movement, including in combat roles, was propagated as part of a wider, political transformation of society towards gender equality, of which the PLA was understood to be a catalyst (Yami, 2005). These insights underscore how the political subjectivities of women fighters are crafted within (rather than outside) the web of familial relationships in which these women are embedded (Friedman, 2018).

Another strand of feminist scholarship, on media representations of female-perpetrated political violence, highlights that familial language and imagery are frequently leveraged to delegitimize the actions of armed women and contain the threat they pose to patriarchal power structures (Gentry and Sjoberg, 2015: 12). Typical media representations depict women soldiers/militants either as victims or as exceptionally heroic fighters who have supposedly stepped outside of their ‘natural’ caregiving roles (Gunaydin, 2023). Familial ties are often deployed to undermine women’s agency and deny their political subjectivity (Tamang, 2017). In contexts as diverse as Chechnya, Indonesia and the former Yugoslavia, armed women are represented as grieving widows (West, 2004); childish daughters (Sunindyo, 1998); and/or devoted sisters accompanying their brothers into battle (Žarkov, 2007) – representations which amplify their personal motives while downplaying their political commitments. Feminist scholarship has also extensively studied how, during armed conflict, motherhood in particular ‘becomes a site of discursive struggle as well as identity politics’ (Žarkov, 2007: 69). In many conflicts, maternalist language and images are leveraged to delegitimize female-perpetrated violence and portray it as irrational and exceptional (Åhäll, 2012; Gentry and Sjoberg, 2015). Yet, recent scholarship also shows that images of armed mothers are strategically deployed by non-state armed groups to promote women’s enlistment (Bayard de Volo, 2012; Loken, 2021).

While existing research examines how familial language/symbolism is leveraged by external observers and by armed groups, relatively few studies have examined how women fighters themselves interpret and respond to gendered norms and identities associated with family, and indeed, how they invest in familial ties. Second, feminist studies successfully interrogate the cultural politics of motherhood; yet, surprisingly few examine how other familial ties, such as sisterly ties, are invoked to rationalize, enable or thwart women’s participation in fighting forces (e.g. Crowley and Sandhoff, 2017). This gap is particularly puzzling given that the burgeoning feminist literature on military culture and identity highlights the importance of constructing ‘brotherhood’ or brotherly ties within armed forces between male soldiers, for example, to enable socialization (Atuk, 2021; Græger 2018; MacKenzie, 2015). Yet, curiously female bonds, including sisterly ties emerging *within* and through military institutions are rarely discussed or recognized as having strategic or political importance – this highlighting the gendered assumptions within the literature around what familial ties matter for understanding armed conflict.

### Material bases of participation

Mobilizing for war and sustaining armed organizations requires labour – material, symbolic and emotional. Feminist scholarship on the material reproduction of armed groups highlights that it is the family that provides the gendered distribution of labour which is indispensable for sustaining war (Catignani and Basham, 2021; Hedström, 2020). This section focuses on what we term ‘the material bases of women’s participation in armed groups’, highlighting the material practices through which family is ‘militarized’ to sustain military operations.

Our article builds on feminist analyses of the political economy of war and militarism (Hedström, 2020; Peterson, 1999). Within this literature, family and familial ties emerge vis-à-vis the broader concept of the ‘household’, understood as a space for social reproduction (Hedström, 2017; Rai et al., 2019). Social reproduction means the ‘labour that goes into reproducing social life’ (Rai et al., 2019: 563). It consists of everyday activities that sustain generations and the social context in which a household exists (Hedström, 2017: 583). Feminist theorizing on social reproduction allows us to capture the gendered labour invested in generating and maintaining the family, and to connect this labour with the production of war.

One strand of feminist scholarship unpacks this connection via a focus on the lived experiences of women who are wives/spouses of soldiers, reservists or private security contractors (Catignani and Basham, 2021; Chisholm, 2022; Hyde, 2016). What these accounts powerfully show is that women’s vital contributions to the social reproduction of the household are indispensable for enabling men to soldier, and furthermore, women’s everyday labour reproduces the very entanglements between family and militarism that legitimize and enable war (Chisholm and Ketola, 2020; Hyde, 2016). As the focus of existing literature is on military spouses, what remains under-explored is the relationship between women’s social reproductive labour (specifically, labour that sustains the family) and women’s participation as *members* of fighting forces. Similarly, while the literature on nationalism recognizes both women’s roles as reproducers of the nation and as fighters, these roles are usually addressed in isolation.

Yet, when women join armed forces, this does not necessarily mean relinquishing gendered responsibilities for social reproduction either within the family or indeed within the military (Hedström, 2020; Parkinson, 2023). Capturing these ongoing responsibilities and forms of gendered labour is crucial for understanding women’s continued embeddedness in pre-existing familial ties as well as the generation of new forms of familial ties through the armed group. Jenny Hedström’s (2020) notion of ‘militarized social reproduction’ is a useful stepping-stone to start examining these connections as it situates women’s everyday labour in both household and the army within the same framework (p. 14). Hedström examines women’s participation in fighting forces not as a separate category of labour, but as interwoven into the social reproductive labour that ‘enables’ war. Hedström’s analysis includes women’s enlistment as fighters, suggesting that conscription is intertwined with other forms of women’s everyday labour. Crucially, she mentions the importance of familial ties: ‘The affective tie of family and community relations ensures that everyone is invested in supporting the revolution. This includes through conscription’ (Hedström, 2020: 8). Our focus is precisely on unpacking and systematically examining such connections between familial ties and women’s participation in armed groups. To do this, the next section develops our conceptualization of familial ties as affective bonds that emerge and transform through war.

## Familial ties as affective attachments

We explore familial ties as affective bonds that are formed and transformed in relation to violence (Ketola, 2023) – and the implications for understanding armed group processes, namely the recruitment and retention of fighters. Our framing builds on what we call ‘feminist theories of the relationality of violence’. This scholarship highlights the complex co-existence of subjectivity, victimhood and violence and uses this analysis to examine wartime and post-war politics (Baines, 2016; Das, 2006; Roy, 2012). Within this scholarship, the realm of family and intimate ties is made and remade through violence (Das, 2006; Matarazzo and Baines, 2021). Building on our discussion of social reproductive labour, we understand familial ties as bonds that do not simply exist, but need to be constantly crafted, cultivated and invested in – a process that Matarazzo and Baines (2021) capture as ‘becoming a family’. Familial ties are affective bonds that are constantly being (re)produced and re-enacted through embodied practices and interactions, such as cooking, caring for others, visiting family members, participating in specific rituals and ceremonies, and so on. In conditions of war, these everyday practices, embedded in broader societal gender norms, become entangled with political violence (Jensen, 2023).

The notion of ties as distinctly ‘affective’ bonds captures our emotional investments in the lives of others and our relationship to others. This includes the affective labour that goes into sustaining such attachments and the various emotions that these ties generate – such as joy, feeling loved, anger, disappointment or fear (Chisholm and Ketola, 2020). In broad terms ‘affect’ can be understood as embodied feelings that are yet to register as emotions, including moods, sensations, dispositions and sensibilities (Anderson, 2017). Drawing on Ahmed (2014), we understand affect and emotions as intimately connected. We are less invested in developing a distinct conceptualization of affect. Instead, we explore how attachments to others are underpinned by and generate specific embodied feelings, and examine what these *do* – what these affects enable (Åhäll, 2019). And specifically, how they may shape women’s participation in war (Dyvik, 2016: 134; Krystalli and Schultz, 2022).

One way to think about ‘what affects do’ in relation to familial ties and women’s participation in armed groups is to note how norms, including gender norms, are affectively felt (Ahmed, 2014). The ‘common sense’ of gender – the taken-for-granted elements of what gender is and how it is to be performed – are powerful precisely because they are not reflected upon but rather register as affects (Åhäll, 2019). Connecting this to the realm of family, what makes familial ties *matter* is not simply the existence of gender norms that structure family as a social institution or the material labour that goes into sustaining the household, but also the affective investments in these ties and in the norms that structure them (Chisholm and Ketola, 2020). For example, in the context of the recruitment of ex-Gurkha soldiers for private security, Chisholm and Ketola (2020) show how the gendered norms around what constitutes a ‘good Gurkha wife’ (pp. 277–278) are affectively felt *and* how these embodied feelings are conditioned by intergenerational and colonial histories of militarism. To understand how women’s participation in armed groups is shaped by family as a militarized social institution, we need to examine affective investments in both the ties that constitute family *and* the gendered norms that *shape* such ties in contexts of war.

Furthermore, capacities to ‘affect and to be affected’ (Anderson, 2014: 9) may enable specific ties to be crafted, including within and through armed groups. A nuanced literature examines bonding between soldiers and the specific practices that armed groups pursue to generate these bonds, including hazing, drills or breaking of existing familial ties. Woodward and Jenkings (2011) highlight the importance of ‘fictive kinship and camaraderie among soldiers’ (p. 253) for constructing the military identities of individual soldiers. Fictive kinship describes the ‘strong emotional bonds’ between soldiers and across groups which are ‘forged through collective endeavour’ and are expressed using familial terms (Woodward and Jenkings, 2011: 260–261). MacKenzie (2015) highlights that a fraternal form of kinship usually emerges within male-dominated military institutions, underpinned by a ‘band of brothers’ myth. Recent scholarship explores further the *affective* dimensions of these bonds and how they shape, for example, practices of socialization and retention (Matarazzo and Baines, 2021; Matfess, 2023). As Krystalli and Schulz (2022) ask in their call to ‘take love and care seriously’: ‘What are the different spaces, forms and relationships in which love shows up within armed groups?’ (p. 18). This points to the need to expand our understanding of what kinds of affective ties matter in analysing armed conflict and armed group processes.

To expand the range of affective ties in focus, our framework conceptualizes a dynamic relationship between war’s violence and familial ties. Baines’ work on political subjectivity and intimate ties is instructive here. Baines (2016) asks us to carefully consider how in conflict ‘some are not only targets of violent events, but are transformed by it in relation to others’ (p. 16). She develops her analysis in the context of the Lord’s Resistance Army (LRA) in Uganda, namely the experiences of women who were abducted as young girls and then endured forced marriage (Baines, 2016). The LRA used this violent, coercive practice to break pre-existing familial ties and generate new forms of relationality and attachments to the armed group. There are several layers to her argument, but most relevant for our discussion is the notion that politics is intricately interwoven with intimate ties – with the attachments and bonds we have to others (Baines, 2016: 13–14). Baines (2016: 125) offers glimpses into how women in the LRA negotiated and reasserted themselves as subjects in the face of violence – by insisting upon, remaking and remembering, their relations and attachments to others. The contexts of our analysis differ significantly – unlike in the context of the LRA in Uganda, the forced recruitment of women and the institutionalization of forced marriage were not prevalent in BiH and Nepal. However, we draw from Baines (2016: 6, 16) the crucial insight that violence may transform subjects *in their relations to others*.

To explore the generative and transformative relationship between war’s violence and familial ties, we construct two frames. First, we use the frame of ‘familial ties as emergent through war’ to capture the kinds of ties that women’s participation as fighters generates within and through the armed group, highlighting how these ties may be expressed in familial terms. What emerges as central here are processes of socialization, including practices of political education, as well as the everyday practices that constitute the life of soldiering or life ‘underground’, such as cooking, cleaning and sleeping (Riley, 2022; Zharkevich, 2019). Through these processes, forms of ‘army family’ are crafted into being (Matarazzo and Baines, 2021). Our exploration highlights both ties that are generated through specific institutional arrangements, such as revolutionary marriage, and in broader terms ties that emerge through participation in the armed group and are expressed in *familial terms*. Importantly, these ties that constitute family ‘underground’ or an ‘army family’ are not isolated from pre-existing familial ties that female fighters may foster and continue to be embedded in (Jensen, 2023; Roy, 2012).

Our second frame, ‘familial ties as transformed through war’ captures how both pre-existing and emergent familial ties are in various ways (re)configured *vis-à-vis* wider transformations in societal gender norms that militarized violence affects. Manipulating and restructuring gendered norms and expectations within the family is central to enabling war. This may involve, for example, the armed group’s efforts to reframe roles and gendered expectations within the family to recruit women (Viterna, 2013) or direct policies that seek to restructure, for instance, the institution of marriage (Giri, 2023). We are interested in understanding how this wider restructuring of gender norms is felt and experienced within the embodied interactions that generate and maintain affective ties. For example, how might ties between daughters and parents be enacted differently, in contexts where women joining the fighting becomes a possibility or even a requirement by the armed group? Or how might revolutionary marriage generate further transformations in the gendered distribution of social reproductive labour within a family?

## Context and methods

To illustrate our argument, we draw on primary data collected during several fieldwork trips to Bosnia (O’Reilly) and Nepal (Ketola) between 2013 and 2022. Both countries have recently experienced armed conflict. The 1992–1995 war in BiH involved a conflict between the Bosnian government and ethno-nationalist rebels (backed by Serbia and Croatia), over the existence and governance of the state. The conflict was rooted in the dissolution of Yugoslavia following a financial crisis, the rise of nationalism and the collapse of consociation mechanisms soon after Yugoslavia’s first multiparty elections in 1990. The ‘People’s War’ in Nepal (1996–2006) was a leftist insurgency, fought by the CPN-M against the government of Nepal, to overthrow the over 200-year-old Hindu Monarchy and replace the existing state with a new democratic platform and secular republic. The insurgency was rooted in historical forms of exclusion that marginalized the vast majority of Nepal’s diverse population, and the concomitant extreme levels of rural poverty.

In both BiH and Nepal, women were permitted or encouraged to join fighting forces during wartime, including combat roles. In Nepal, women constituted approximately 30 percent of PLA members – the armed wing of the CPN-M that rapidly evolved from a small group of fighters into a highly organized military (Ogura, 2008; Yadav 2016). Women from rural areas were at the forefront of the Maoist movement, participating as fighters, political cadres, artists and medics (Yadav 2016). In BiH, women constituted approximately 5 percent of ARBiH members (Hadžiahmić 2011) – initially formed as a multi-ethnic force to fight against Bosnian Serb (and later Bosnian Croat) ethno-nationalist rebellion. Women ARBiH members undertook frontline combat duties and non-combat roles (e.g. administration and logistics).

We have chosen to bring these case studies into a dialogue, first, because our fieldwork and subsequent analysis of interviews from both contexts revealed important patterns in how familial ties structured women’s participation in fighting forces. Second, in both BiH and Nepal women’s participation in armed groups was largely voluntary, even if taking place amid highly coercive circumstances. Women generally volunteered to undertake military roles in the ARBiH^2^ and forced recruitment of women did not predominate in either context.^3^

Our main source of information consists of in-depth, semi-structured interviews with women who joined the ARBiH or PLA.^4^ Maria completed 27 interviews with women who participated in the military defence of Sarajevo as ARBiH members. Hanna undertook 13 interviews with women who participated in the Maoist movement as whole-time members living underground during the war. Our respondents discussed their journeys into the fighting forces and their experiences of armed struggle. What emerged from these narratives was the centrality of familial ties to understanding women’s participation in fighting forces. Familial ties emerge and transform within and through conditions of war. And these familial ties both enabled and constrained women’s participation in various ways. Delving deeper into interview narratives, we shed light on the complex relationship between familial ties and women’s political subjectivities as fighters. For example, entering a revolutionary marriage not only signifies commitment to political struggle but also generates new familial ties and responsibilities that must be negotiated for women to continue to participate in fighting forces.

In examining two case studies, our goal is not to generate a ‘rigid and systematic comparison’ (Krystalli and Schulz, 2022: 10) or ‘paired comparison’ (Fujii, 2017: 664). Instead, we bring these cases into dialogue and use examples from both contexts to illuminate the complex, dynamic relationship between familial ties and women’s participation in fighting forces in conflicts where forced recruitment of women specifically does not predominate. Our approach echoes the extended case study methodology used in critical and feminist IR research. We constructed (rather than selected) our cases through ‘dynamic interaction’ with feminist theory, and produced our theoretical framework and typology of militarized familial ties following a series of extensions that allowed us to move from the situated knowledge we gained through fieldwork to an understanding of the social processes and forces that shaped these ties (Lai and Roccu, 2019).

Our feminist, post-structuralist approach views knowledge-building as a ‘dialectical process’ which involves listening to women who joined fighting forces and understanding the ‘subjective meanings they attach to their lived experiences’ of participation (Tickner, 2006: 21). We adopted a reflexive approach, by building our theoretical framework from the interview material we gathered in each context and allowing our understanding of the relationship between militarized familial ties and women’s participation in armed groups to emerge incrementally through different stages of research.

Following in-depth fieldwork, we read and re-read our interview transcripts and field notes and identified examples where our interlocutors highlighted how familial ties shaped, or were shaped by, their participation in fighting forces. Then, we zoned in on two key processes pursued by armed groups – recruitment and retention – and produced rich descriptions of how familial ties were generated by, and transformed through, these processes in both contexts. We situated these rich descriptions within the wider context of each armed conflict, noting how familial ties were shaped by wider transformations in gender norms that war generates. Next, we brought our case studies into dialogue, using feminist IR scholarship on the cultural, material and affective bases of participation as a lens through which to make sense of our data. By engaging in ‘continuous dialogue between researcher and participants, and between fieldwork material and theoretical reflection’ (Lai and Roccu, 2019: 80), we have built a theoretical framework that responds to the complexities of each case yet is also flexible enough to be adapted and extended to other contexts of armed conflict where forced recruitment of female combatants does not predominate.

## Analysis: familial ties and women’s participation in armed groups

### Emergence of familial ties through women’s participation in armed groups

We focus first on the familial ties that war’s violence generates. We examine how new ties emerge within and through women’s participation in fighting forces. We also explore how women continue to invest in pre-existing familial bonds, including ties which were severed or strained by war’s violence.

#### Biljana and Lejla’s story: cultivating sisterly ties and embodying resistance to ethno-nationalism

In Maria’s interviews with women who joined the ARBiH, several participants reflected on how familial ties emergent through the armed group were crucial for the development of political subjectivity. In Sarajevo, women’s participation in armed struggle was often prompted by a desire to defend their families, neighbourhoods and their city against ethno-nationalist aggression and secure the survival of BiH as a unified, multicultural state. Joining the ARBiH – initially constructed as a multi-ethnic force^5^ – allowed women to forge new familial ties that enabled them to resist the forces of ethno-nationalism.

Here, we focus on the narratives of Biljana and Lejla, who joined an ARBiH combat unit. Both were young when they enlisted. Although volunteers, both enlisted in response to highly coercive circumstances. Lejla’s enlistment was sparked by the sudden severance of familial ties. Serb forces killed Lejla’s father and detained her mother early in the war, and she remained separated from her mother for two years. Finding herself alone, Lejla decided she had ‘no alternative’ but to enlist. Biljana’s enlistment was prompted by a rising sense of insecurity. Her father was captured in the war’s early months, and she feared that her parents’ ‘mixed marriage’ (Catholic and Orthodox Christian heritage) placed her family at heightened risk. Intermarriage between ethno-religious groups was common in pre-war Sarajevo and symbolized the ‘brotherhood and unity’ of all peoples who lived in socialist Yugoslavia (Simmons, 2002). Yet, across ex-Yugoslavia, rising nationalism and the onset of war meant such marriages increasingly became ‘taboo, something to be eliminated, something disturbing’ (Morokvasic-Muller, 2004: 142). As Biljana reflected: ‘I thought [enlistment] was a good way to protect my family, because we were living in an environment where the Muslim population represented the majority’ (Interview 1). By contributing to the collective defence of multi-ethnic Sarajevo, one of Biljana’s goals was to dissolve any ambiguity associated with her family’s identity.

Both women eagerly explained that the close friendship they crafted within the ranks took on a sisterly quality. They forged a close bond, spending more time away from home sleeping in the forest, and so on and became inseparable – ‘like flesh and a nail’, according to Lejla. Biljana described Lejla as being ‘like my sister, because I spent more time with her than with my own sisters’. These reflections demonstrate the importance of their ‘sisterhood’ in helping to sustain Biljana and Lejla through periods of loss and extreme hardship. Moreover, both made striking connections between the cultivation of this sisterly tie and the crafting of political subjectivity linked to armed struggle. For instance, Lejla remarked that despite the pair’s Muslim and Orthodox heritage, they would share ‘even the tiniest piece of bread’. Biljana recalled that Lejla was a constant source of support on several occasions when she experienced hostility and threats to life based on her ascribed ethnicity (even as a fighter, she was viewed by some as an ‘enemy’).^6^ Lejla revealed that when her sick mother was released via a prisoner exchange, it was Biljana who carried Lejla’s mother on her back through a secret underground tunnel connecting besieged Sarajevo to Bosnian-held territory west of the city. The pair then transported her on foot 10 km or so across Sarajevo ‘under the constant shooting of the snipers, shells, bullets’ so that she could be admitted to hospital (Interview 1).

These acts of solidarity and mutual support – ranging from the apparently mundane to the extraordinarily courageous – not only strengthened their sisterly bond but also demonstrated their commitment to defending Sarajevo’s multicultural and multi-ethnic character. Crafting this tie was both a personal and political choice. Biljana and Lejla refused to view each other through an ethno-nationalist prism of ‘ethnic difference’, and instead forged a bond so close that they regarded each other as sisters. Their narratives demonstrate that familial ties and political subjectivity are closely intertwined in contexts of armed conflict. At a time when ethno-nationalist and religious differences were violently imposed, and sexual violence against women was widespread, investing in this sisterly bond can be read as a manifestation of resistance against extremism and patriarchal domination (see Tumbas, 2022). Cultivating this bond allowed Biljana and Lejla to express and inhabit a political subjectivity that rejected constructions of ethnic difference imposed upon them.

#### Dil Sari’s story: ties emergent through revolutionary marriage and inhabiting motherhood as a fighter

In Hanna’s interviews with women who participated in the Maoist movement, several women reflected on their experiences of marrying a comrade and having a child while still engaged in the armed struggle. Revolutionary marriages (*janabadi bibaha*, literally, people’s marriages) were relatively common among Maoist cadres, with the movement tacitly propagating the idea of ‘love marriage’ in wider policies regulating the libidinal economy of war (Zharkevich, 2019: 129). We focus on how this wider restructuring of family was felt and enacted in everyday interactions, spotlighting how the ties that emerged through revolutionary marriage shaped women’s participation.

Here we focus on the story of Dil Sari, a political leader in the Maoist movement. She reflected on her decision to leave her one and a half year old daughter with family members to return underground. Dil Sari grew up in Thabang, a village designated as the ‘Maoist Capital’ during the war. She was first active in the Maoist cultural group and then the student organization, going fully underground soon after the launch of the People’s War as a young adult. She progressed through the ranks, becoming a prominent leader in the party’s women’s wing. Dil Sari explained how she had met her husband when active in student politics – ‘he was mobilized by me’ Dil Sari pointed out. After five years spent underground they married, seeking the party’s approval. Dil Sari’s marriage started a series of complex negotiations when what she designated as her ‘personal life and emotional relationships’ were to co-exist with her political work. These negotiations intensified when two years later their daughter was born.

Dil Sari shared how she made the decision to leave her daughter with family members, this crystallizing in the question of whether to join a major attack against the army:

I struggled a lot in my heart . . . It is a big thing for a mother to decide to leave her child. I consulted my husband, about leaving my daughter. ‘Leave her wherever you please’ he said. When talking to my mother-in-law, she said yes. I cried as I carried my daughter to her grandmother. My husband said, ‘If you are going to cry, go back. Some women have even abandoned their six-month-old babies. If you don’t like it, go back’. (Interview 2)

As Dil Sari said, ‘It is a big thing for a mother to decide to leave her child’. So much is packed into that statement. One aspect that Dil Sari narrated was her sisters’ advice about breastfeeding – how her daughter was too young for her to leave. This illustrates the endurance of social norms around biological reproduction, and what was expected from Dil Sari ‘as a mother’. Yet these gendered forms of relationality were narrated in conjunction with another set of responsibilities, those to the party and to her comrades. For Dil Sari, a planned major attack emerged ‘as the right time to leave’ as the party had made her a commander of medics, and she explained that ‘the whole set would be ruined’ unless she participated.

The subject position of being a ‘mother’ and a ‘fighter’ is a potent combination (Gentry and Sjoberg, 2015). Through narrating her husband’s comments, Dil Sari invoked Maoist policies encouraging women to leave their children with relatives or in communal childcare and return to their lives as whole-timers (Zharkevich, 2019). Through these policies, Dil Sari’s decision to leave her daughter was legitimized as a possible – or even as a preferred choice – *within* the movement. Consequently, we gain insights not only into how maternal logics are utilized by armed groups to authorize women’s participation (Loken, 2021), but also how women within armed groups draw on and reflect on these logics when deliberating on their continuous contribution. But what also emerges is the gendered affective labour and responsibility that is placed on Dil Sari who is effectively left to make the decision on her own – ‘If you don’t like it, go back’.

When thinking about political subjectivity as interlaced with motherhood, one way to start is to pause when Dil Sari tells us that she ‘struggled a lot in her heart’. The way she pursued her political subjectivity as a *Maobaadi*, mobilizing other women, made her acutely attuned to the gender norms underpinning her decision (Yadav, 2016: 98). As she explained later in the interview: ‘Feminist thinking dominated me. I thought of not getting married’. And yet, highlighting this political commitment should not mean writing out the affective attachments to her daughter that Dil Sari so strikingly narrated. As she explained, when walking with her comrades, she saw women carrying their children. ‘When I see a woman with a baby on the road. I say to them that my child is like that too’. Dil Sari’s reflections illustrate how being a mother *and* a Maoist are not separate facets of subjectivity that can be selectively inhabited depending on the situation. Perhaps part of what the ‘struggle in the heart’ entailed for Dil Sari was a constant ‘stitching together’ of the political struggle and life with its multiple conflicting attachments (Jensen, 2023: 465). This ‘boundary work’ (Jensen, 2023: 478) is not something that just happens but requires immense affective labour and reconfiguration of ties, including pre-existing ones.

Another striking moment in Dil Sari’s interview concerned the question of who took on the labour of caring for her daughter. Dil Sari initially left her daughter in her care mother-in-law’s care, but she was quite old and found it hard to manage. Dil Sari explained how her cousin took over:

[S]he took care of her *like her own daughter. Although she was a cousin, she took care of her like [she was my] sister*.

The same sister was present in the interview, listening closely when Dil Sari narrated experiences that were part of both of their lives. This included Dil Sari remembering how her sister comforted the baby when she could not sleep due to stomach problems. The woman commented on how it had been difficult, and she had no idea how to do it. It was Dil Sari’s sister who took on the affective and physical labour of caring for the child. And it was precisely such forms of gendered labour – performed predominantly by other women – that allowed Dil Sari and others to keep pursuing the struggle. It is in this sense that ‘militarized social reproduction’ (Hedström, 2020) manifests here, enabling not only men’s soldiering (Hyde, 2016) but the participation of women in armed struggle. In the process of militarization, the tie between Dil Sari and her cousin is reconfigured, taking on new meanings and importance. By capturing this subtle transformation, we gain valuable insights into *how* familial ties may enable (or thwart) women’s continuous participation. The next section expands on this insight, zooming into the question of how ties may be re-configured within and through women’s participation in fighting forces.

### Transformation of familial ties through women’s participation in armed groups

As new ties emerge and pre-existing ties endure, affective bonds are transformed vis-à-vis the wider shifts in societal gender norms that armed conflict sets in motion. Our research encounters in BiH and Nepal highlighted how women’s participation in fighting forces sparked transformations in familial ties or, more broadly, in societal norms around the institution of family. We examine how this wider restructuring of gender norms is felt and experienced within the everyday, embodied interactions that generate and maintain affective ties.

#### Anesa’s story: Joining the ARBiH and becoming a son rather than a daughter

Anesa was a school-leaver when she joined the ARBiH as a fighter. Her enlistment story touches upon important themes including the restructuring of gender relations and affective ties within the family as crucial for enabling women’s participation. Moreover, it highlights the intensity of emotions generated by the transformation of familial ties by war’s violence.

Describing her enlistment, Anesa highlighted two deliberative encounters that signalled important shifts in gender relations within her family and community. The first encounter – meeting a commander at her local recruitment station – highlights that, by becoming a fighter, she transgressed established norms surrounding the ‘appropriate’ place of women in war:

. . . [A]fter having watched my friends, my peers from school . . . join the defence of Bosnia, one day I got ready and went to our unit so that I would sign up too. . . . I asked the commander if I too could join. He said, ‘Alright. Would you like me to give you a ladle or a pen?’ I said, ‘Neither of those. I want to sign up to the Army to be a fighter’. That surprised him. (Interview 3)

The commander was ready to accept Anesa as a cook or administrative officer and expressed surprise when she insisted on taking up a close combat role. No combat exclusion policy was in operation; his reaction reflected and reinforced essentialist gender norms associating combat with masculinity. Anesa’s insistence on taking up a ‘masculine’ role challenged cultural norms which assigned women subordinate roles in armed struggle.

Anesa’s second encounter – sharing her enlistment decision with her parents – illustrates how wartime changes in societal gender relations are (re)interpreted and negotiated within those webs of affective ties that constitute family. When war commenced, Anesa was living with her father, mother and three sisters. Her father was the only household member obliged to join Bosnia’s military defence. Anesa’s enlistment both challenged and reinforced idealized masculinities and femininities associated with being a ‘daughter’ or a ‘son’:

When I got back home, I told my mum and dad I signed up. Mum fainted; she fainted from fear. How come I, a woman, could sign up to the Army of Bosnia & Herzegovina! At that moment, my father came close to me, hugged me, and said, ‘This is my son!’ He began to cry. He said, ‘I do not have a son. I have the four of you, but you are my son’. When I signed up, he began to cry with happiness, there, because I showed courage by signing up to the Army of Bosnia & Herzegovina. (Interview 3)

Since women were barred from the Yugoslav National Army for several decades, Anesa’s mother struggled to reconcile the subject position of woman and fighter. Her father also struggled to connect femininity with soldiering. Instead, he interpreted Anesa’s enlistment decision as equivalent to her taking up the role of a son in their family. In this encounter, the recruitment of a female soldier activated the (re)interpretation and negotiation of gender norms and identities within her family and prompted a transformation of familial bonds.

Anesa’s narrative powerfully highlights that when affective ties are transformed, this may generate intense emotions which can work to bolster (or undermine) women’s participation in fighting forces. Her mother physically collapsed in shock on hearing Anesa’s news, fearful of what this decision would entail. In contrast, Anesa’s father responded with intense pride, interpreting his daughter’s decision as courageous. Despite their contrasting reactions, both parents’ affective responses reinforced the sedimented norm that it is the responsibility of a *son* rather than a *daughter* to become a fighter in wartime. This illustrates how gendered norms are affectively felt and how such affective investments condition transformations of familial ties during war (Åhäll, 2019: 43; Ahmed, 2014).

Anesa’s account points to the influential role that parents may play in facilitating or thwarting armed groups’ voluntary recruitment of female fighters. While her mother’s beliefs and values regarding gender roles and divisions of labour may have constituted a barrier to Anesa’s enlistment, her father’s acceptance and support served as an important enabling factor. In many Bosnian families, the mother is viewed as a ‘pillar’ of the household, who dominates quietly in the private sphere without challenging the authority of the father (Spahić-Siljak and Kosović, 2012). Like many societies, there is often an uneven distribution of familial power and privilege with the father often viewed as head-of-the-family. In Anesa’s case, her father’s willingness to accept (and embrace) his daughter’s decision, helped clear the way for Anesa to enlist as a fighter. Affective ties to the family were a crucial factor shaping Anesa’s decision and experience of recruitment.

#### Dolma’s story: withdrawing participation to secure family’s survival

In thinking about the transformation of societal gender norms that war affects, it is important to examine how such transformation may co-exist with enduring gendered responsibilities within the family (Yadav, 2016). In Dolma’s story, the call for women’s liberation and her continuing embeddedness in affective ties and responsibilities to sustain her family are narrated as oppositional to the extent that she periodically withdraws her participation in the party, returning several years after the peace agreement. It would be possible to write Dolma’s story as one where familial ties function as a barrier to women’s participation in armed groups. Yet, it is more interesting to view its subtleties where Dolma’s connectedness to the struggle is not simply severed but rather reconfigured, allowing her to foster ties with her family, secure their survival and then return to political activity.

Dolma joined the Maoist movement at a young age from Ramechhap, a rural district that became a Maoist stronghold after the movement spread from mid-Western hills to Eastern Nepal. She had six younger siblings, and her older sister had already married and left home, this leaving her as the ‘eldest younger sister’. She first became a dancer in the Maoist cultural group and then soon joined the PLA. For Dolma, as for many other young people in Nepal, participating as a whole-timer meant living underground, walking long distances at night, and not having regular contact with family members. Dolma shared an encounter with her parents when, during a ceasefire, she came home for two weeks’ leave. Her parents had heard rumours that she had been killed.

I was locked inside the house. They said, ‘You came back alive, don’t go now. We shall die of worry when you go to fight again’. Comrades came to get me . . . I ran away and reached Doramba [nearest larger municipality]. There was a Maoist program. Father and sister’s children reached Doramba after me. Then Baba said, ‘I raised you. We have grown old. Children have also come to bring you back. If you don’t return home, I won’t return home either’. After Baba said this, I left the party. (Interview 4)

When Dolma shared more, further dense negotiations emerged between her comrades and parents:

During the war, comrades also came to take me. However, father and mother did not let me go. My friends from the party told me to come too . . . Parents used to scream at my friends. I drowned in the feelings of my parents. I couldn’t go.

Dolma’s story powerfully illustrates the affective dimension of how familial ties constrain participation – with her striking expression of how she ‘drowned’ in her parents’ feelings. Dolma’s narration of these exchanges reminds us how the capacity to affect and to be affected is central to how gender norms are reiterated and consolidated (Åhäll, 2019; Chisholm and Ketola, 2020). The parents’ feelings of worry for Dolma’s life – and the concomitant urgency to keep her at home become intermingled with Dolma’s responsibilities towards maintaining the family life. In pleading for Dolma to stay, her father invokes not only her responsibilities to look after the parents (‘we have grown old’) but also her responsibilities as the ‘eldest-younger sister’, telling her that her nieces and nephews have ‘come to bring you back’. Dolma continues to affectively invest in ties to her parents and siblings, even after she has *already* joined the armed struggle, this continued embeddedness starting to shape her journey in the Maoist movement.

Yet, Dolma’s decision to withdraw her contribution needs to be situated within the broader material context of the crisis of social reproduction and ongoing war (Rai et al., 2019). As Dolma explained later: ‘*I left the party to look after my house because there was not enough food in the family*’. What is striking is how these responsibilities *shift* over time. At the time of our interview, 16 years after the peace agreement, Dolma had just been elected as a representative of local government in her rural municipality, having been selected as a candidate for the main Maoist party, CPN (Maoist Centre). She explained,

I joined the party only after I could see my family earn and eat . . . My brother has grown up now . . . I put my brother and daughter-in-law in charge of the house, and I became active in the party again . . . I also stood up as a female candidate in the recent election. I won.

By examining both the endurance *and* shifts in gendered responsibilities for social reproduction over time, we can illuminate how decisions around participation are not ‘one-off’ moments, but rather continuous processes of deliberation that in Dolma’s case continue in the war’s aftermath.

### Discussion and typology of militarized familial ties

Our analysis of in-depth interviews with women who joined fighting forces during the wars in BiH and Nepal offered crucial insights into the situated experience of armed conflict. Since they capture everyday encounters and embodied interactions within the violent and coercive context of armed conflict, these narratives offer a critical lens through which to unpack the intricate connections between familial ties and women’s participation in armed groups. Biljana’s and Lejla’s narration of their sisterly tie, for instance, powerfully illustrates this intricate web of relationality and how it shapes experiences of participating. When reading their story, one might ask: why did these women invest in this specific tie?

One way to answer this is to point to the violent severing of ties between Lejla and her parents through forced displacement, and the concomitant desire to cultivate a close bond with a comrade who became like her sister. Another way is to focus on the manipulation of family by ethno-nationalist leaders, and Biljana’s wish to address the (gendered) forms of insecurity that are generated when ethnic ‘otherness’ is constructed as a threat. Both answers point to the complex web of familial relationships that the pair were embedded in, which shaped their decisions about whether and how to participate in the military defence of Sarajevo. These readings (always contingent and not final) illustrate that both the cultural and the material bases matter in understanding women’s participation in armed groups; as does the affective – fear, loneliness and other emotions that are generated by war’s violence. The sisterly bond that Biljana and Lejla forged is not reducible to a strategic relationship or tactical choice (cf. Utas, 2005) but rather is embedded in a broader web of affective familial bonds emergent through and transformed by war.

When conflict is viewed from the standpoint of women who participated in armed struggle, the importance of familial ties for enabling war is thrown into sharp relief. Our analysis reveals that the connection between familial ties and women’s participation in fighting forces is co-constitutive and mutually reinforcing, with each element functioning as both a product and an enabling condition of the other. In one direction, we see that the affective bonds that constitute family in armed conflict situations are generated by, and transformed through, armed group processes in which women are embedded (such as recruitment and retention of fighters). In the other direction, familial ties both enable and constrain the enlistment, withdrawal and re-enlistment of women in fighting forces.

**Diagram 1.**
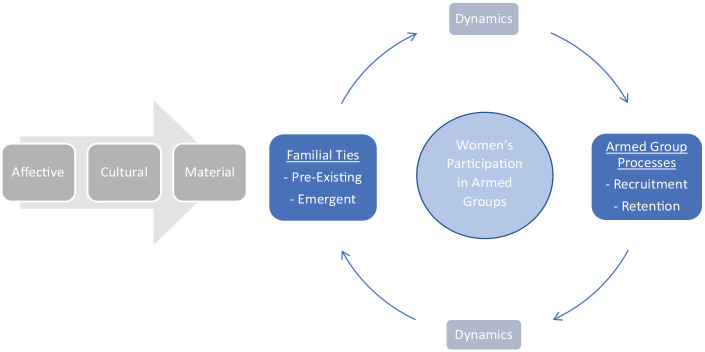
Familial Ties and Women’s Participation in Armed Groups

To open avenues for further research, we offer a systematic framework for analysing the co-constitutive relationship between familial ties and women’s participation in armed groups (Diagram 1). This framework highlights the dynamic interaction between familial ties and processes pursued by armed groups (of which we take as examples the recruitment and retention of fighters). Our diagram shows how familial ties shape, and are shaped by, women’s participation in armed groups. This diagram is based on our ‘top-down’ analysis of existing literature combined with ‘bottom-up’ analysis of the experiential accounts of women fighters in BiH and Nepal.

On the left-hand side of Diagram 1, we highlight the affective, cultural and material bases of women’s participation in fighting forces, to illustrate the crucial significance of *family* to understanding armed conflict. On the right, we foreground the dynamic relationship between *familial ties* and women’s participation in fighting forces. Here, we spotlight three main elements which are crucial for unpacking this relationship: (a) types of familial ties, (b) processes of armed groups and (c) dynamics of change.

We distinguish between ‘pre-existing’ and ‘emergent’ types of familial ties – the first category captures ties existent at the time of joining, while the latter describes ties that are crafted within and through the armed group. In terms of processes of armed groups, our analysis focuses on two key processes – the recruitment and retention of fighters. However, there is considerable potential to extend the scope to cover other processes, such as the socialization of fighters. Regarding the dynamics of change, we distinguish between (1) the emergence and (2) transformation of familial ties within conditions of war – to draw attention to the interaction between the types of familial ties and processes of armed groups. We view this dynamic interaction as two-directional, whereby armed group processes have an impact on the familial ties that are crafted and/or transformed in war, and vice versa.

We also introduce a new typology – of *militarized familial ties* – for organizing and analysing data on how familial ties shape and are shaped by women’s participation in fighting forces. Our typology is grounded in extensive reading of literature and careful empirical analysis of fieldwork interviews. It captures the two-directional nature of the relationship between familial ties and armed group processes. This typology outlines five distinct ways in which pre-existing and emergent familial ties shape and are shaped by women’s participation, focusing specifically on processes of recruitment and retention:

1. Familial ties that emerge through participation in armed struggle can be a motivating factor to join an armed group.
2. Familial ties that emerge through participation in armed struggle shape the retention of fighters.
3. Pre-existing familial ties can be transformed by joining an armed group.
4. The continued embeddedness of fighters in pre-existing familial ties conditions the retention of fighters.
5. Pre-existing familial ties may be transformed through retention.

These five statements are not an exhaustive list but rather allow researchers to systematically capture the dynamic relationship between familial ties and women’s participation in armed groups (as we have done in cases of BiH and Nepal).^7^

The four stories we discussed above illustrate one or more of these statements. Lejla and Biljana’s story, for example, demonstrated that the prospect of cultivating new familial ties may be an important factor underpinning the decision to join an armed group – by enlisting in the ARBiH, Lejla forged a close sisterly bond with Biljana following the violent severing of her pre-existing ties to her parents (statement 1). Dil Sari’s story highlighted the ties that emerged through revolutionary marriage (tie to her daughter, and her husband) and the ties to her comrades, all of which conditioned her decision-making regarding whether to sustain her participation or alternatively withdraw from armed struggle (statement 2). Anesa’s story of enlisting in the ARBiH highlighted that familial ties may transform following recruitment – for example, ties between a daughter and her parents were reconfigured as Anesa took up the role of a son (statement 3). Dolma’s embeddedness in pre-existing familial ties conditioned her continued participation as she withdrew her participation to take on responsibilities for social reproduction to secure her family’s survival (statement 4). Dil Sari’s decision to sustain her participation in the armed group generated a transformation in pre-existing familial ties, as her cousin took on the reproductive labour of caring for Dil Sari’s daughter (statement 5). These stories also illustrate the two-directional relationship between familial ties and armed group processes that we capture in our typology. Dil Sari’s story, for instance, shows that familial ties were generated through her participation in the PLA, *and* her participation also prompted a transformation in pre-existing ties.

## Conclusion

Our article has explored the critical nexus between familial ties and armed conflict. We have developed a novel framing of *militarized familial ties* that captures the cultural, material and affective bases of women’s participation in fighting forces. This framing conceptualizes familial ties as affective bonds that emerge and transform within and through conditions of war. This dynamic framing has allowed us to systematically capture and analyse the co-constitutive relationship between familial ties and women’s participation in armed groups.

Our framework and original empirical analysis make crucial contributions to the wider IR and Conflict Studies scholarship that examines social ties in contexts of armed conflict. We argue that this literature should conceptualize family as a gendered social institution that is constituted by ties that are distinctively affective in nature. Familial ties *matter* not only because family is a powerful social institution. They matter also because the women participating in armed groups continue to cultivate and invest in these ties and the norms that structure them.

This argument has crucial implications for understanding armed group processes of recruitment and retention. First, we show that familial ties are not merely or even primarily a barrier to women’s participation but also function as an enabling factor. Second, and delving deeper, we argue that there is a distinct affective quality to *how* familial ties condition – enable and constrain – women’s participation in armed groups. The affective quality of these ties creates contradictory demands for women in fighting forces, with women making decisions regarding whether to join, sustain their participation or leave armed groups in relation to these familial bonds.

Based on our novel theoretical framework and original empirical analysis, we introduced a new *typology* which captures the co-constitutive relationship between familial ties and women’s participation in armed groups. Our typology allows researchers to build systematic knowledge of the relationship between familial ties and women’s participation in armed groups, and compare findings across contexts using a reflexivist approach (Lai and Roccu, 2019). Since our typology details how familial ties are produced through and shape processes of recruitment and retention, it also offers possibilities for understanding the conditions under which women are likely to join, sustain and/or withdraw their participation in armed groups. We encourage other researchers to expand the typology to cover other armed group processes, including socialization. Since our typology is based on detailed analysis of two different contexts where women generally volunteered to join armed groups, it can be reflexively extended to other conflict situations where forced recruitment of women does not predominate.

At its core, our argument contributes to feminist IR scholarship on women’s participation in armed groups by offering new avenues to understand how familial ties and political subjectivities intersect. We highlight the intricate ways in which political subjectivities are cultivated through the affective ties that emerge in/through war, and pre-existing ties in which women continue to be embedded even after joining (rather than merely in opposition to such ties). We hold that when exploring political subjectivities emerging through participation in armed groups, feminist analysis cannot shy away from thinking deeply about familial ties as affective bonds. This does not need to mean reproducing problematic constructions of women fighters as always already associated with the ‘maternal’, ‘familial’ or ‘domestic’ realm. Rather, what we have unravelled here, for example, via Dil Sari’s reflections of how she ‘struggled in her heart’, is the constant work that goes into crafting, sustaining and reconfiguring familial bonds within conditions of war, a form of affective labour that women whose narratives we have centred were all in different ways engaged in.

## Notes

1. These attachments are embedded in the broader norms that structure ‘family’ in distinct ways depending on the social context.

2. During the war in BiH, male citizens aged 18–55 were subject to compulsory military service. Women were not obliged, but could volunteer, to undertake military service.

3. At the height of its recruitment drive, the CPN-M adopted a policy of ‘one house one Maoist’ which constituted a clear form of forced recruitment.

4. To ensure anonymity, we use pseudonyms to identify participants.

5. As Hoare (2004) notes, over time, the ARBiH became a predominantly Muslim/Bosniak force.

6. For example, one comrade threatened to kill Biljana after his relative was killed by Serb forces.

7. We recognize that the types of familial ties, processes of armed groups and dynamics of change that operate within a conflict are context-specific.
